# A bibliometric analysis of complement in IgA nephropathy from 1991 to 2022

**DOI:** 10.3389/fphar.2023.1200193

**Published:** 2023-07-27

**Authors:** Yun Guo, Haiqiang Zhang, Xueqing Yu

**Affiliations:** ^1^ The First Clinical Medical College, Guangdong Medical University, Zhanjiang, China; ^2^ Department of Nephrology, Guangdong Provincial People’s Hospital (Guangdong Academy of Medical Sciences), Southern Medical University, Guangzhou, China; ^3^ BGI-Shenzhen, Shenzhen, Guangdong, China; ^4^ Guangdong-Hong Kong Joint Laboratory on Immunological and Genetic Kidney Diseases, Guangzhou, China

**Keywords:** IgA nephropathy, complement, citespace, vosviewer, bibliometrics

## Abstract

**Introduction:** IgA nephropathy is a common glomerular disease on a global scale, which has resulted in significant economic burdens. The complement system plays a vital role in enhancing the efficacy of antibodies and phagocytic cells in eliminating microbes and damaged cells, and promoting inflammation. Complement activation has been found to contribute to the progression of various renal diseases, including IgA nephropathy.

**Methods:** In this study, a thorough analysis was conducted on publications related to complement in IgAN from 1991 to 2022, retrieved from the Web of Science Core Collection and Scopus database. The analysis focused on various aspects such as annual publications, country, institution, author, journal, keywords, and co-cited references, utilizing Citespace and Vosviewer.

**Results:** A total of 819 publications were obtained, and while there were slight fluctuations in annual publications, an overall upward trend was observed. China, Japan and the United States were the leading countries in terms of publications, with China having the highest number of publications (201). Collaborative network analysis revealed that England, University of Alabama Birmingham, and Robert J Wyatt were the most influential country, institution, and author, respectively, in this field of research. Furthermore, the analysis of references and keywords indicated that complement activation contributes to IgAN, and immunosuppression in IgAN are a hot topic of research.

**Discussion:** This study identifies current research hotspots and advanced tendencies in the study of complement in IgAN, providing scholars with crucial directions in this research area.

## Introduction

Immunoglobulin A nephropathy (IgAN), also known as Berger’s disease, is the most common primary glomerulonephritis worldwide, affecting 2–10 per 100,000 person-years (McGrogan et al., 2011; Jarrick et al., 2019). The symptoms of IgA nephropathy can range from mild to severe, and in some cases, there may be no symptoms at all. Common symptoms include hematuria, proteinuria, edema, high blood pressure, and decreased kidney function, accounting for ∼30% of the terminal renal failures in patients within 10–20 years after diagnosis (Knoop et al., 2013).

The pathogenesis of IgA nephropathy involves a complex interplay of genetic, environmental, and immunological factors, leading to the formation of IgA1 immune complexes and activation of the complement system, resulting in podocyte and tubulointerstitial injury (Lai, 2012).

The Complement System is a network of soluble and cell membrane proteins that function in a coordinated manner to activate the classical, alternative, and lectin pathways (Noris and Remuzzi, 2013). The classical pathway is triggered by the binding of C1q to immune complexes, while the lectin and alternative pathways are activated by the association of C3b, properdin, mannose-binding lectin (MBL), or ficolins with microorganism-associated molecular patterns or carbohydrate structures present on damaged cells. Upon activation of any of these pathways, convertases are formed, leading to the cleavage of C3 and C5 proteins and the formation of the Membrane Attack Complex (C5b-9). The resulting active fragments, including C3a, C3b, iC3b, C3dg, C4a, C4b, and C5a, bind to their respective complement receptors, triggering biological responses (Merle et al., 2015). The alternative pathway is consistently maintained in a state of low-level activation. To prevent unregulated activation, the complement system is subject to regulation by specific proteins such as factor H, factor I, and complement receptor 1. These proteins expedite the decay of the convertases or facilitate the cleavage of activation fragments. Disorders arising from genetic mutations or a deficiency of regulatory proteins can result in insufficient or excessive complement activation. This may initiate or perpetuate various pathological conditions, including autoimmune and renal diseases (Hajishengallis et al., 2017).

## Materials and methods

### Search strategy

A search for publications was carried out by utilizing the Web of Science Core Collection and Scopus database. The search criteria employed were “TS = (complement) and (iga nephropathy) or (immunoglobulin A nephropathy) or (iga glomerulonephritis) or (Berger’s disease)” for the period from 1991 to 2022. The main types of publications were articles and reviews, with the language of the literature limited to English. Data files obtained from Scopus must undergo conversion to Web of Science format in Citespace prior to utilization.

### De-duplication of data

The process of data de-duplication was carried out in two distinct stages. Firstly, a manual de-duplication method was implemented, in which the publications were examined and filtered based on their title, authorship, journal source, publication date, and other relevant information. This step ensured that irrelevant publications were eliminated and accurate de-duplication was achieved. Secondly, the publications obtained in the first step were imported into Citespace to run the duplicate removal function. Document types of article, review, editorial material, meeting abstract were retained. The advantage of this function, aside from removing any duplicated records, is that its output files are arranged by the year of publication. Citespace can take advantage of this order to speed up the subsequent processes such as time slicing.

### Proofreading of data

The normalization of publication data involved merging synonyms for multiple words that held the same meaning. Suppose nodes A and B need to be consolidated into node A, firstly, right-click on node A and select it as the primary alias; secondly, right-click on node B and assign it as the secondary alias; thirdly, repeat these steps for any additional pairs needed to be merged; finally, re-run the process GO.

### Extraction of data

The publication data were extracted through the employment of Citespace and Vosviewer. The dataset includes, but is not limited to, the number of publications, date, countries, institutions, authors, references, and keywords.

### Citespace

**FIGURE 1:**
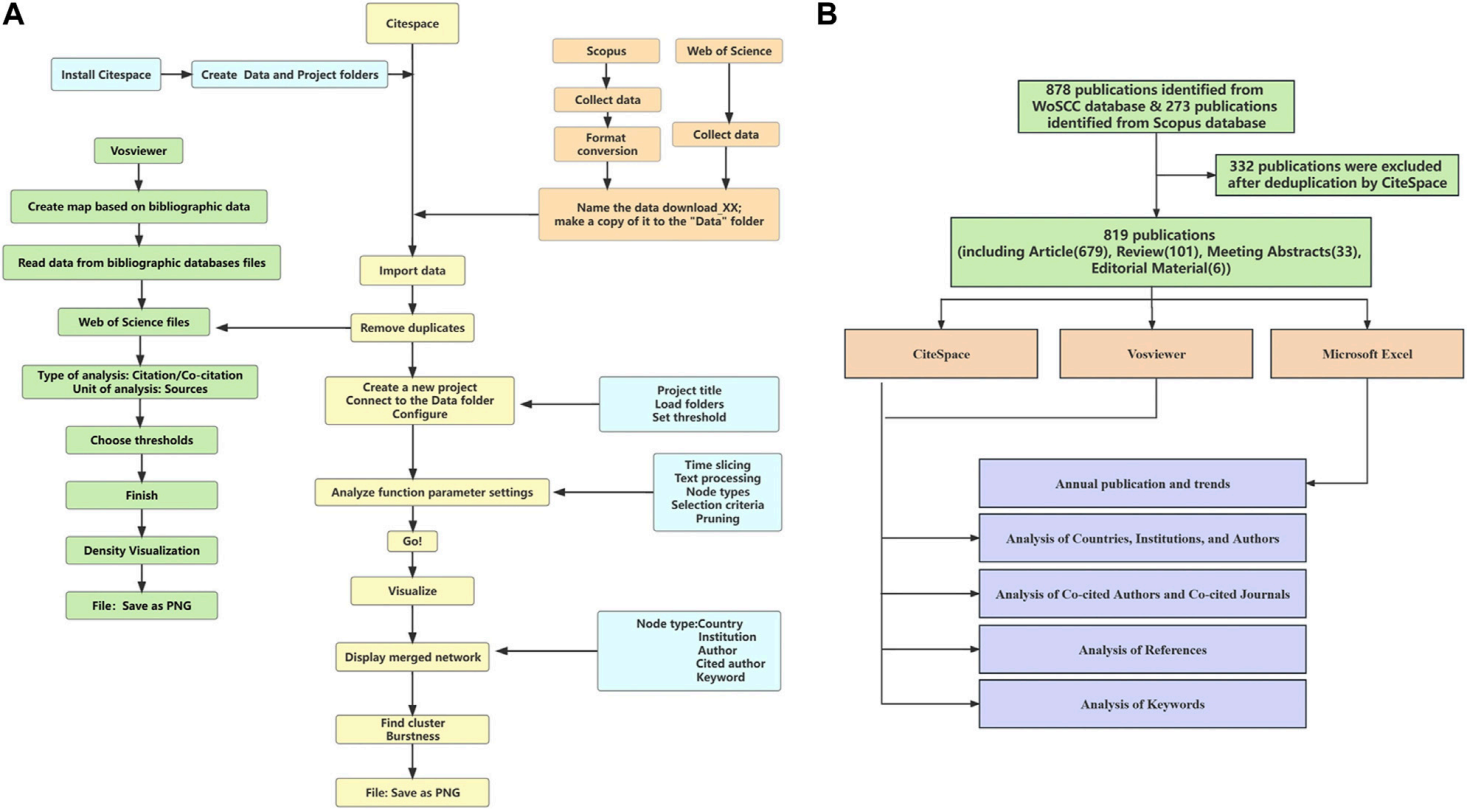
Flowchart. **(A)** Flowchart of using Citespace and Vosviewer. **(B)** Flowchart of searching publication.

Citespace (version 6.2.R3) is a Java-based software application developed by Chaomei Chen of Drexel University in Philadelphia, PA. Its purpose is to provide visualization of patterns and trends in scientific publications (Figure 1A). Citespace allows for the visualization of knowledge structures and hotspots within research fields, as well as the tracking of research frontiers. It facilitates understanding of scientific developments and enables scientific predictions (Chen and Chen, 2005; Chen and Song, 2019). In this study, the specific parameters of Citespace were set as follows: 1) Time Slicing: From January 1991 to December 2022; Years Per Slice: 1; 2) Term Source: Title, Abstract, Author Keywords, Keywords Plus; 3) Node Types: Author, Institution, Country, Keyword, Category, Reference, Cited Author, and Cited Journal; 4) Selection Criteria: g-index: k = 10; Top N = 50; 5) Pruning: Pathfinder, Pruning sliced networks (Chen et al., 2012).

### Vosviewer

Vosviewer (version 1.6.19) is a software application in the Java programming language designed to facilitate the creation and visualization of bibliometric networks. Vosviewer can be used to construct networks of scientific journals based on bibliographic database files such as Web of Science and Scopus files (Figure 1A). In addition, Vosviewer provides three visualizations of a map: The network visualization, the overlay visualization, and the density visualization. Zooming and scrolling functionality allows a map to be explored in full detail, which is essential when working with large maps containing thousands of items (van Eck and Waltman, 2010).

## Results

### Annual publication trends

**FIGURE 2:**
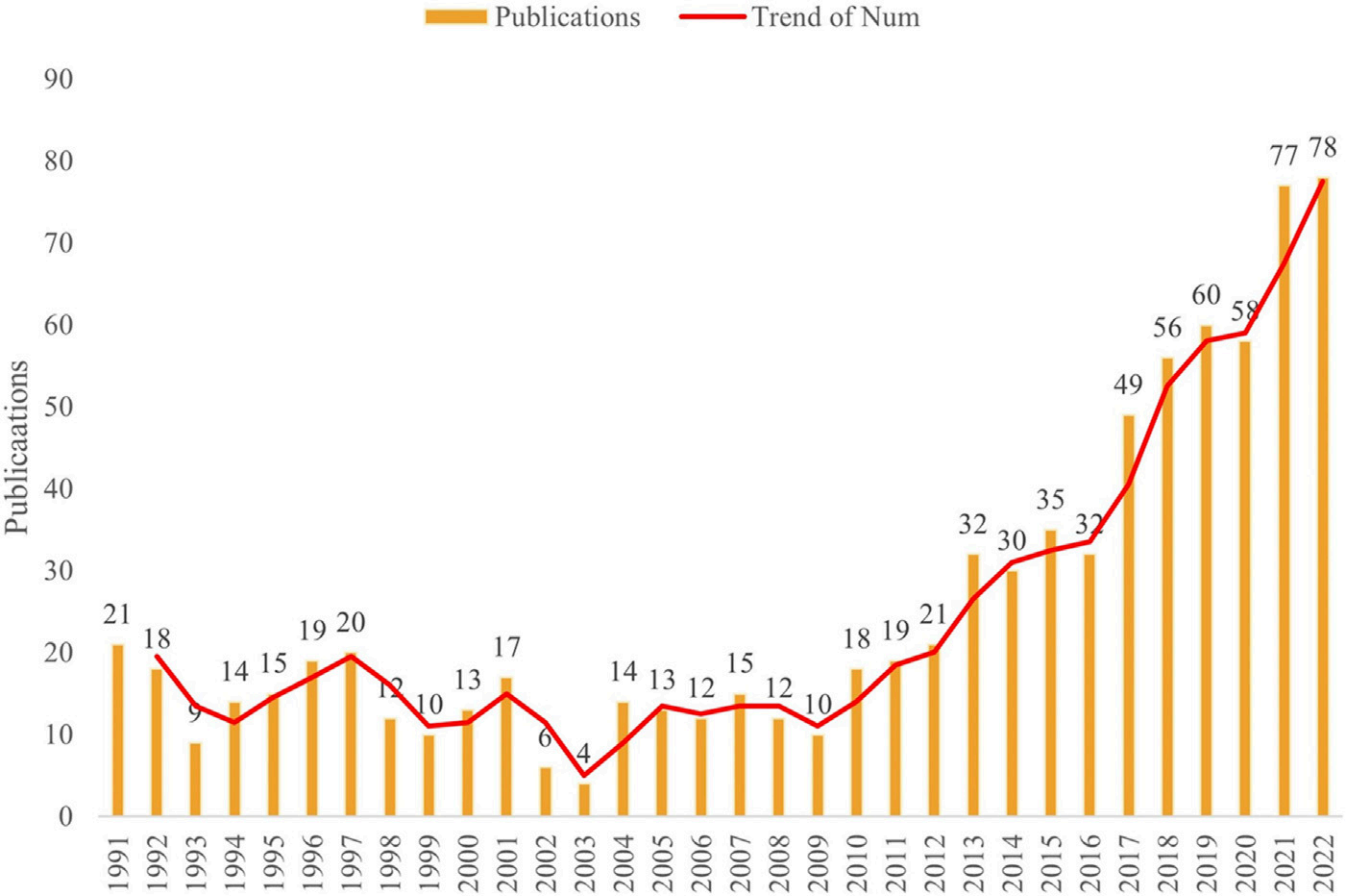
Annual publication of complement in IgAN.

This study comprised a total of 819 publications, encompassing 679 articles, 101 reviews, 33 meeting abstracts, 6 editorial materials (Figure 1B). As shown in Figure 2, the number of publications related to complement in IgAN showed a fluctuating upward trend. The publications in 2016–2022 showed an obvious upward movement.

### Analysis of country

**FIGURE 3:**
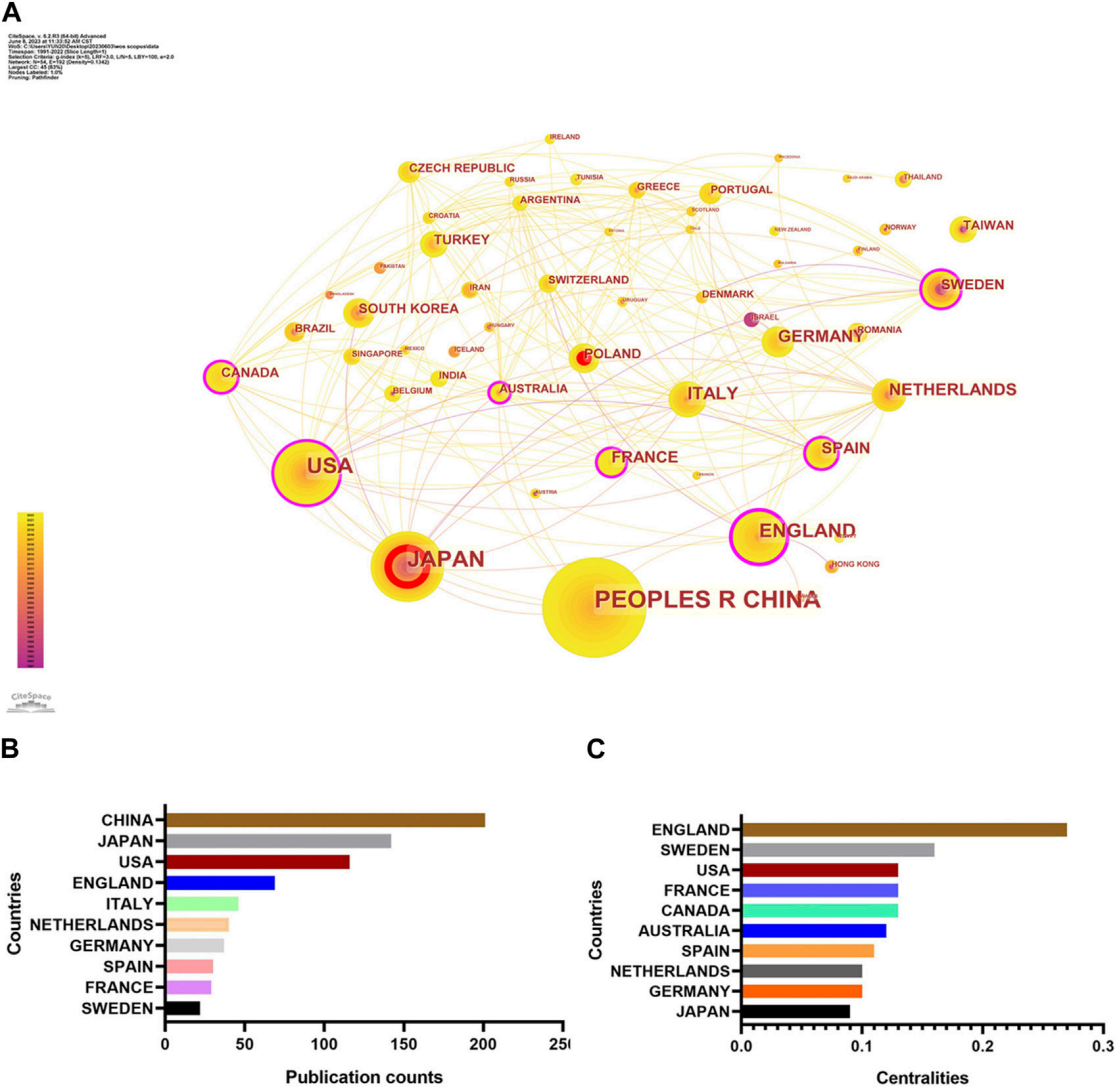
Contribution of different countries to complement in IgAN. **(A)** Map of countries related to complement in IgAN. **(B)** Top 10 countries in terms of publication counts. **(C)** Top 10 countries in terms of centralities.

A total of 54 countries have documented their findings on the topic of complement in IgAN. As shown in Figure 3, the highest number of publications has been recorded in China with 201, followed by Japan with 142 and the United States with 116. In China, the mechanism of complement in IgAN has been the subject of study since 1992, with a yearly increase in the number of publications. In Japan, research on this topic has been conducted since 1991. Similarly, the United States began studying this topic in 1991. In terms of international collaboration, countries with centrality values greater than 0.1 include the England (0.27), Sweden (0.16), the United States (0.13), France (0.13), Canada (0.13), Australia (0.12) and Spain (0.11). England displays the highest degree of international cooperation, followed by Sweden and United States, in terms of research collaboration. This longstanding trend of international collaboration has been evident since the early stages of research in England.

### Analysis of institutions

**FIGURE 4:**
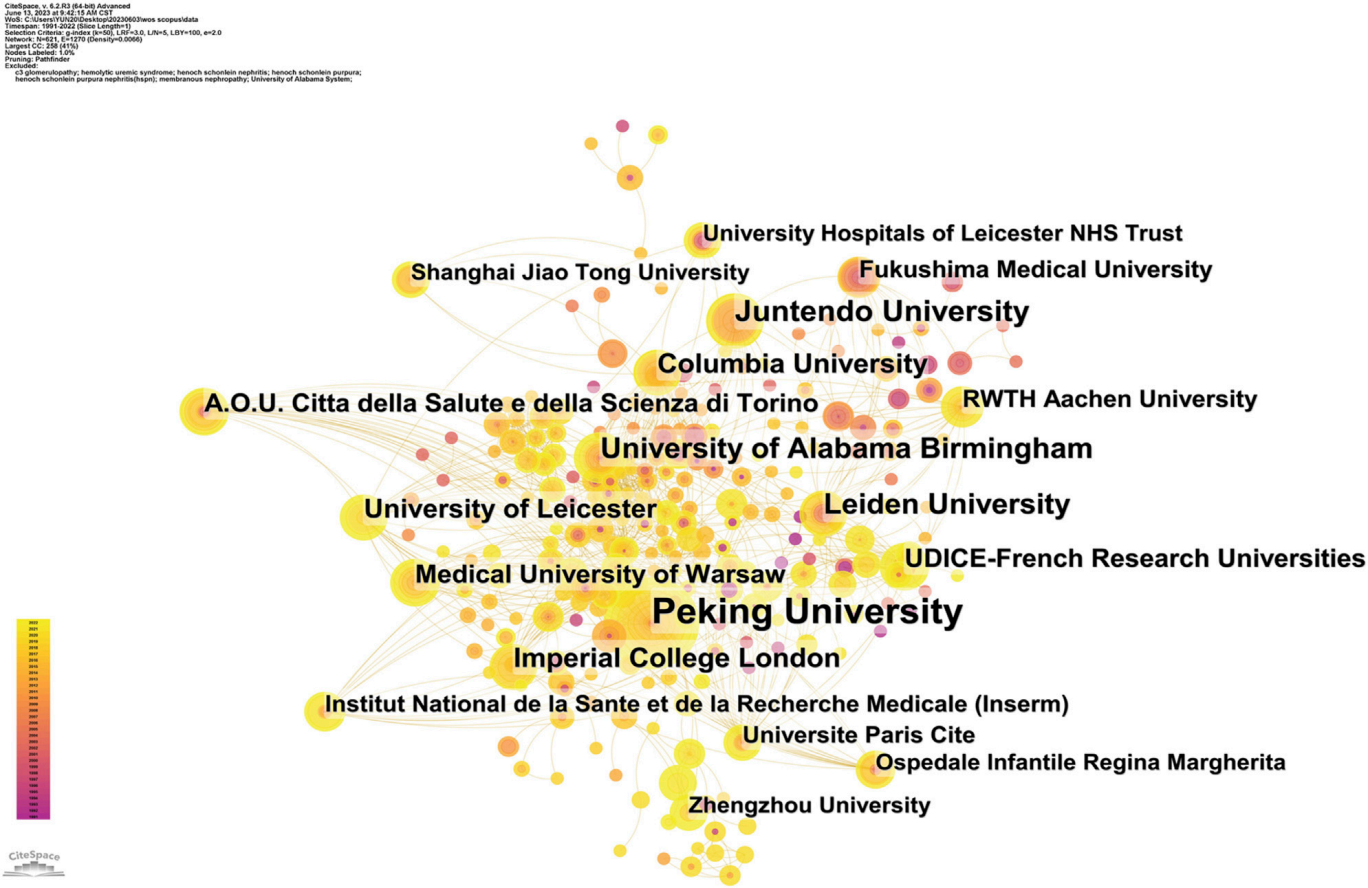
Contribution of different institutions collaborations related to complement in IgAN.

The analysis of the institutions was conducted using Citespace. The graph nodes represent individual institutions. The size of each node is directly proportional to the number of publications produced by the institution. The connections between the nodes depict the extent of cooperation between the institutions, and the color of the connection signifies the duration of the cooperation. The thickness of the connection symbolizes the intensity of the cooperation. Peking University published the greatest number of academic publications (58), followed by Juntendo University (26), University of Alabama Birmingham (24), Leiden University (24) (Figure 4) (Table 1). Peking University has been conducting research on complement in IgAN since 1992. The institution’s latest publication posits that complement is involved in the development of arteriolar microangiopathic lesions in IgAN. This finding may be associated with the circulating complex containing Gd-IgA1 (Li et al., 2022). Juntendo University and University of Alabama Birmingham initiated research on complement in IgAN in 1991, while Leiden University began research on this topic in 1999. The latter institution proposed that factor H-related proteins in IgA nephropathy may contribute to enhanced complement activation (Floege and Daha, 2018; Renner et al., 2022). The top 4 institutions of centrality are University of Alabama Birmingham, Peking University, Juntendo University, and Columbia University. Columbia University has undertaken extensive research on complement in IgAN since 2011. In the latest publication, it collaborates with other institutions posit that complement activation plays a pivotal role in the pathogenesis of IgA vasculitis with nephritis (Hastings et al., 2022).

**TABLE 1:** Institutions related to complement in IgAN.

Rank	Institution	Count	Rank	Institution	Centrality
1	Peking University	58	1	University of Alabama Birmingham	0.08
2	Juntendo University	26	2	Peking University	0.06
3	University of Alabama Birmingham	24	3	Juntendo University	0.05
4	Leiden University	24	4	Columbia University	0.05
5	Imperial College London	20	5	Imperial College London	0.03
6	Columbia University	19	6	University of Leicester	0.03
7	University of Leicester	18	7	Medical University Warsaw	0.03
8	Medical University Warsaw	15	8	RWTH Aachen University	0.03
9	A.O.U. Citta della Salute e della Scienza di Torino	15	9	Fukushima Medical University	0.03
10	UDICE-French Research Universities	15	10	Chinese Academy of Medical Sciences	0.03

### Analysis of author and co-cited author

**FIGURE 5:**
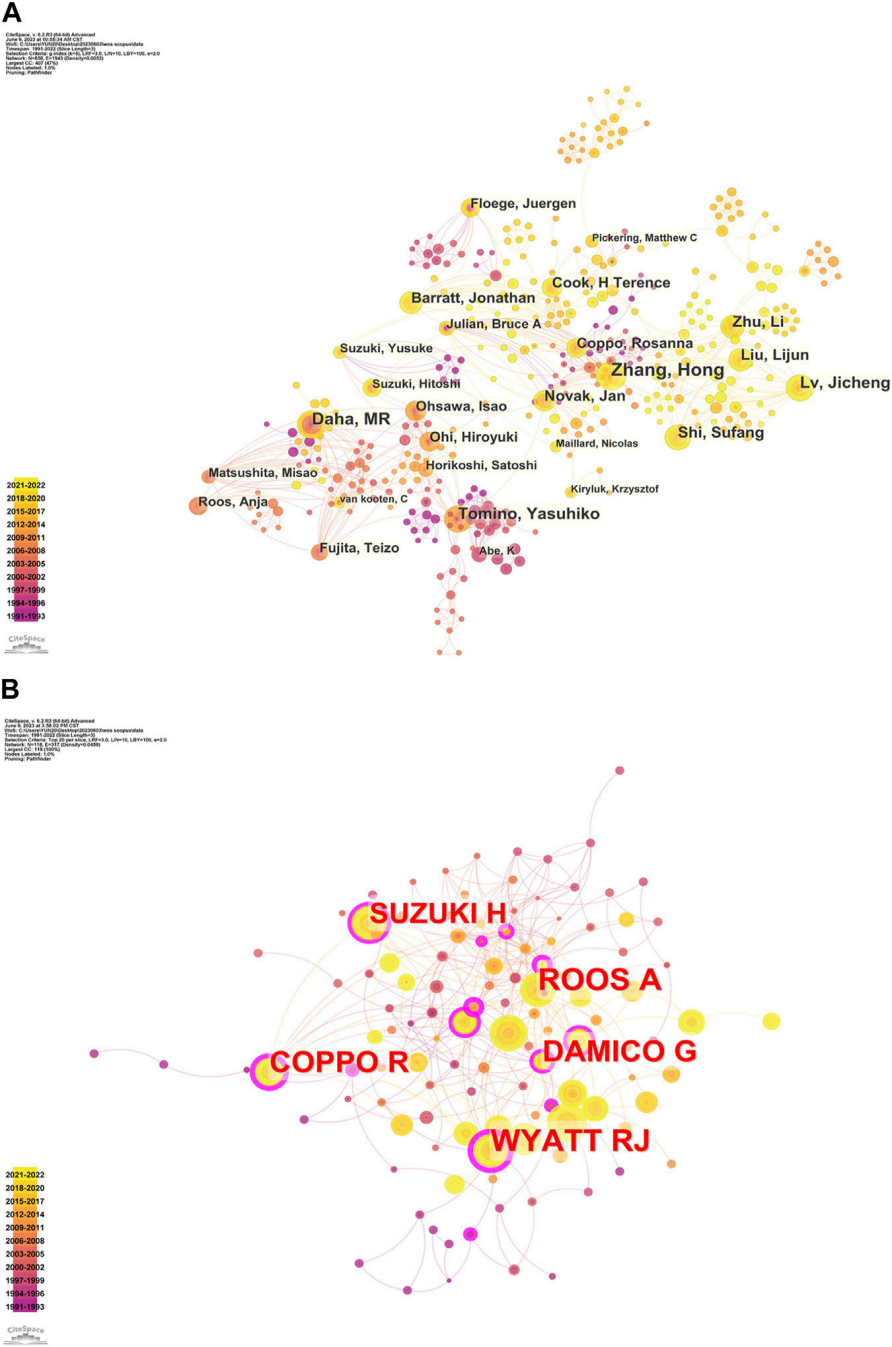
The collaboration network of authors. **(A)**The collaboration network of authors. **(B)** The collaboration network of co-cited authors.

A comprehensive visual analysis of the author has been carried out using Citespace, with a total of 824 authors involved in the study of complement in IgAN (Figure 5) (Table 2). The analysis revealed that Hong Zhang has the highest number of publications (51), followed by Mohamed R Daha (38), Yasuhiko Tomino (33) and Jicheng Lv (31). The connecting lines between the nodes in the network diagram signify the collaborations between the authors, highlighting the fact that the authors with the highest number of publications have collaborated more frequently with their peers in recent times. The co-citation analysis of the authors also revealed noteworthy findings, with Anja Roos having the highest number of publications (173), followed by Robert J Wyatt (165), Rosanna Coppo (154), and G D'Amico (141). The top 5 co-cited authors for centrality were Robert J Wyatt (0.54), Yasuhiko Tomino (0.41), Morita Endo (0.29), Rosanna Coppo (0.24), G D’Amico (0.19). Robert J Wyatt is a Pediatrics professor at University of Tennessee Health Science Center, who has made substantial and noteworthy contributions to the field of IgA nephropathy. Rosanna Coppo is a Nephrology professor at the Regina Margherita Hospital, who has made significant contributions in the field of glomerular diseases in both children and adults, with a particular focus on IgA nephropathy and IgA vasculitis (Barbour et al., 2022).

**TABLE 2:** Authors related to complement in IgAN.

Rank	Cited author	Count	Rank	Co-cited author	Count
1	Hong Zhang	51	1	Anja Roos	173
2	Mohamed R Daha	38	2	Robert J Wyatt	165
3	Yasuhiko Tomino	33	3	Rosanna Coppo	154
4	Jicheng Lv	31	4	G D'Amico	141
5	Sufang Shi	27	5	Hitoshi Suzuki	131
6	Li Zhu	26	6	Kar Neng Lai	108
7	Lijun Liu	25	7	Ali G Gharavi	103
8	Jan Novak	23	8	Mario Espinosa	101
9	H Terence Cook	22	9	Nicolas Maillard	100
10	Jonathan Barratt	21	10	Krzysztof Kiryluk	99

### Analysis of journals

**FIGURE 6:**
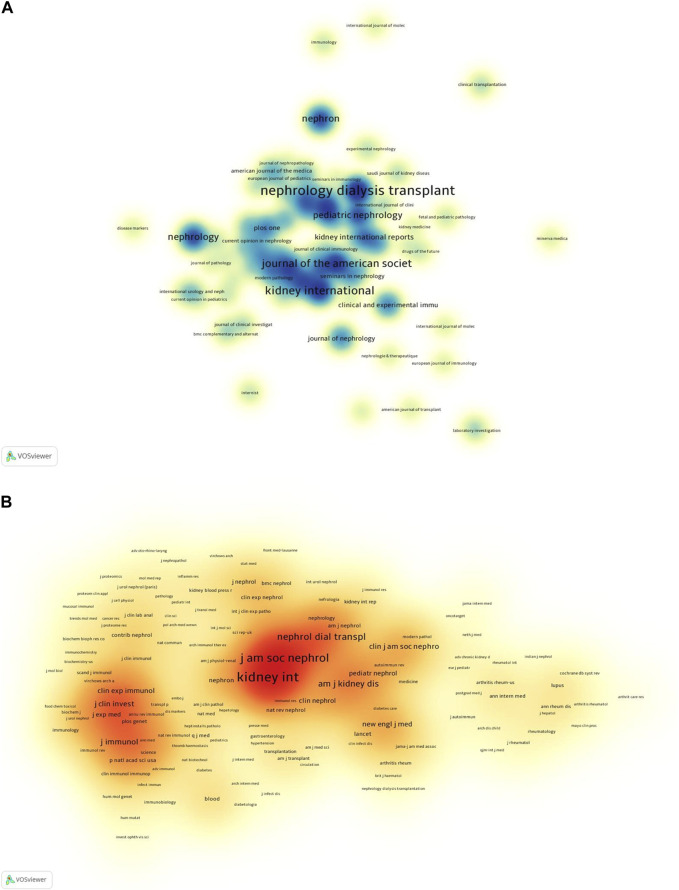
Visualisation of journals related to complement in IgAN. **(A)** Density map of cited journals. **(B)** Density map of co-cited journals.

The analysis of journals for citation and co-citation was performed using Vosviewer, as illustrated in Figure 6. According to the cited journals (Table 3), Kidney International (KI) (120) was found to be the most cited journal, followed by Journal of the American Society of Nephrology (JASN) (74), and Pediatric Nephrology (36). Of the top 10 most frequently cited journals, six were categorized as Q1, three were categorized as Q2, and the remaining two as Q3 in the Journal Citation Reports (JCR), with Kidney International (KI) having the highest impact factor (IF).

**TABLE 3:** Citation journals of complement in IgAN.

Rank	Journal	Citation	TLS	JCR	IF
1	Kidney International	120	341	Q1	18.998
2	Journal of the American Society of Nephrology	74	590	Q1	14.981
3	Pediatric Nephrology	36	233	Q2	3.652
4	Nephrology Dialysis Transplantation	35	298	Q1	7.186
5	Frontiers in Immunology	28	211	Q1	8.787
6	Kidney International Reports	25	134	Q1	6.234
7	American Journal of Kidney Diseases	23	125	Q1	11.072
8	Nephron	23	118	Q2	3.457
9	Molecular Immunology	22	165	Q3	4.174
10	Plos One	14	115	Q2	3.752

Note: TLS, total link strength; JCR, journal citation reports; IF, impact factor.

Regarding co-cited journals, as indicated in Table 4, Kidney International (KI) (2,738) was identified as the most frequently co-cited journal, followed by JASN (2002) and Nephrology Dialysis Transplantation (1,257). Among the top 10 most frequently co-cited journals, seven were classified as Q1 in the JCR and the remaining three as Q2, with New England Journal of Medicine having the highest IF.

**TABLE 4:** Co-citation journals of complement in IgAN.

Rank	Journal	Citation	TLS	JCR	IF
1	Kidney International	2,738	208,954	Q1	18.998
2	Journal of the American Society of Nephrology	2002	178,097	Q1	14.981
3	Nephrology Dialysis Transplantation	1,257	124,380	Q1	7.186
4	American Journal of Kidney Diseases	1,003	91,530	Q1	11.072
5	Journal of Immunology	688	44,052	Q2	5.43
6	Journal of Clinical Investigation	566	39,888	Q1	19.477
7	Clinical Journal of the American Society of Nephrology	557	67,436	Q1	10.624
8	Clinical and Experimental Immunology	518	34,087	Q2	5.732
9	New England Journal of Medicine	513	67,782	Q1	176.082
10	Pediatric Nephrology	443	51,367	Q2	3.652

Note: TLS, total link strength; JCR, journal citation reports; IF, impact factor.

### Analysis of co-cited references

Based on the analysis conducted using Citespace, it was revealed that among the top 10 most cited references, consisting of four reviews and six articles, as depicted in Table 5. The most cited article authored by Anja Roos, which posited that glomerular deposition of MBL and L-ficolin in IgAN was associated with more pronounced histologic damage (Roos et al., 2006).

**TABLE 5:** Co-cited references of complement in IgAN.

Rank	First author	Journal	Citation	Year	Burst	Doi
1	Anja Roos	J AM SOC NEPHROL	152	2006	0	10.1681/ASN.2005090923
2	Nicolas Maillard	J AM SOC NEPHROL	100	2015	13.44	10.1681/ASN.2014101000
3	Ali G Gharavi	NAT GENET	87	2011	0	10.1038/ng.787
4	Robert J Wyatt	NEW ENGL J MED	87	2013	5.8	10.1056/NEJMra1206793
5	Hitoshi Suzuki	J AM SOC NEPHROL	83	2011	4.22	10.1681/ASN.2011050464
6	Daniel C Cattran	KIDNEY INT	82	2009	0	10.1038/ki. 2009.243
7	Mario Espinosa	CLIN J AM SOC NEPHRO	75	2014	7.71	10.2215/CJN.09710913
8	Anja Roos	J IMMUNOL	68	2001	5.84	10.4049/jimmunol.167.5.2861
9	Ian S D Roberts	KIDNEY INT	68	2009	4.84	10.1038/ki. 2009.168
10	Hernán Trimarchi	KIDNEY INT	68	2017	18.35	10.1016/j.kint. 2017.02.003

**FIGURE 7:**
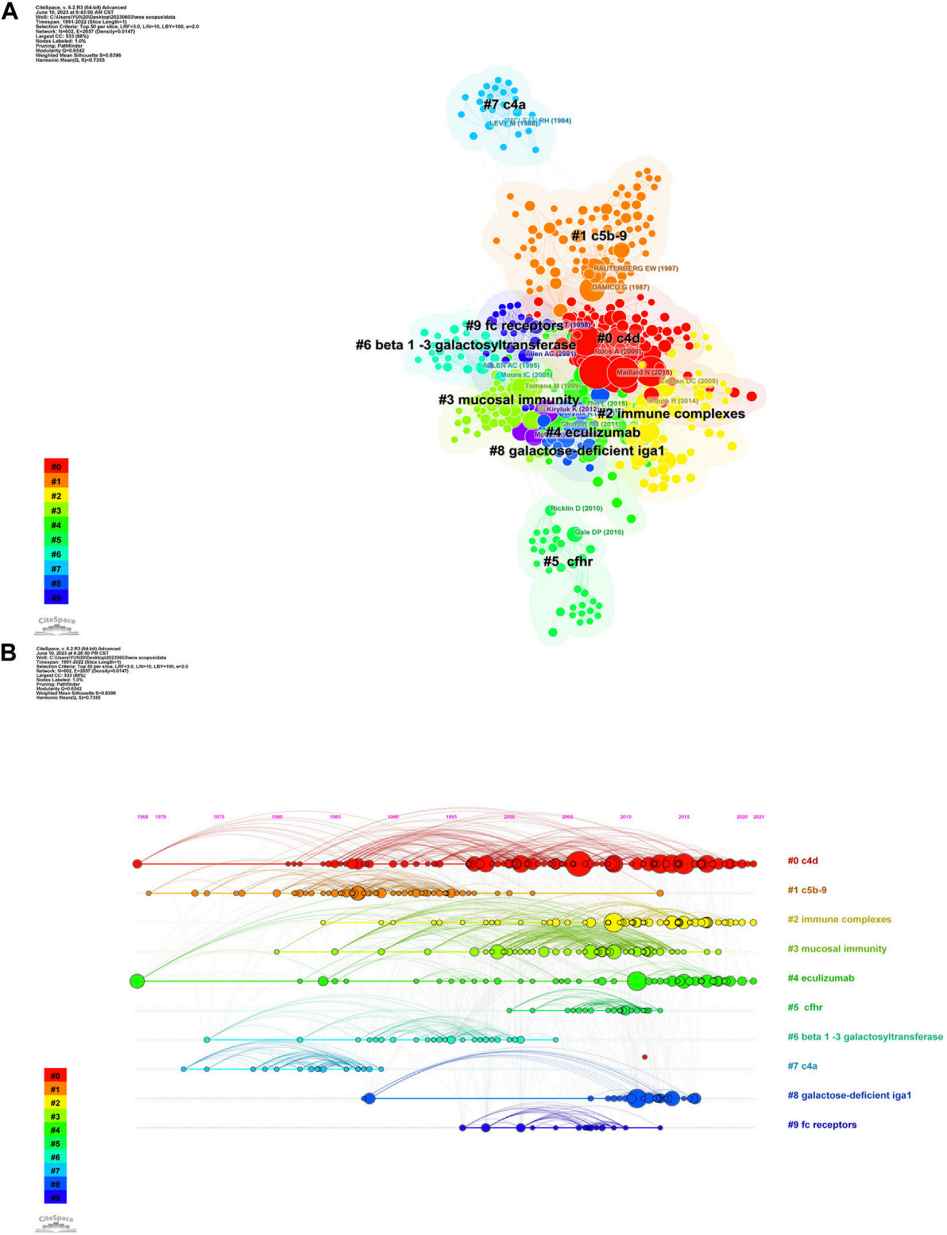
Visualisation of co-cited references. **(A)** The cluster analysis of co-cited references. **(B)** Timeline graph of co-cited reference.

The Citespace software divides entities into clusters, with each cluster representing a distinct concentration of disciplines and specializations (Figure 7). To perform cluster analysis on the co-cited references, a log-likelihood ratio was utilized, resulting in the identification of 10 clusters. These clusters exhibit a modularity of Q = 0.6542, a weighted mean silhouette of S = 0.8398, and a harmonic mean of (Q, S) = 0.7355. The segmented clusters consist of the following: #0 c4d, #1 c5b-9, #2 immune complexes, #3 mucosal immunity, #4 eculizumab, #5 cfhr, #6 beta 1-3 galactosyltransferase, #7 c4a, #8 galactose-deficient iga1, and #9 fc receptors. The timeline illustration of co-cited references reveals that the primary research areas for complement and IgA nephropathy encompassed c4d and c5b-9. Moreover, the current research trends for complement and IgAN involve immune complexes and eculizumab.

### Analysis of keywords

**FIGURE 8:**
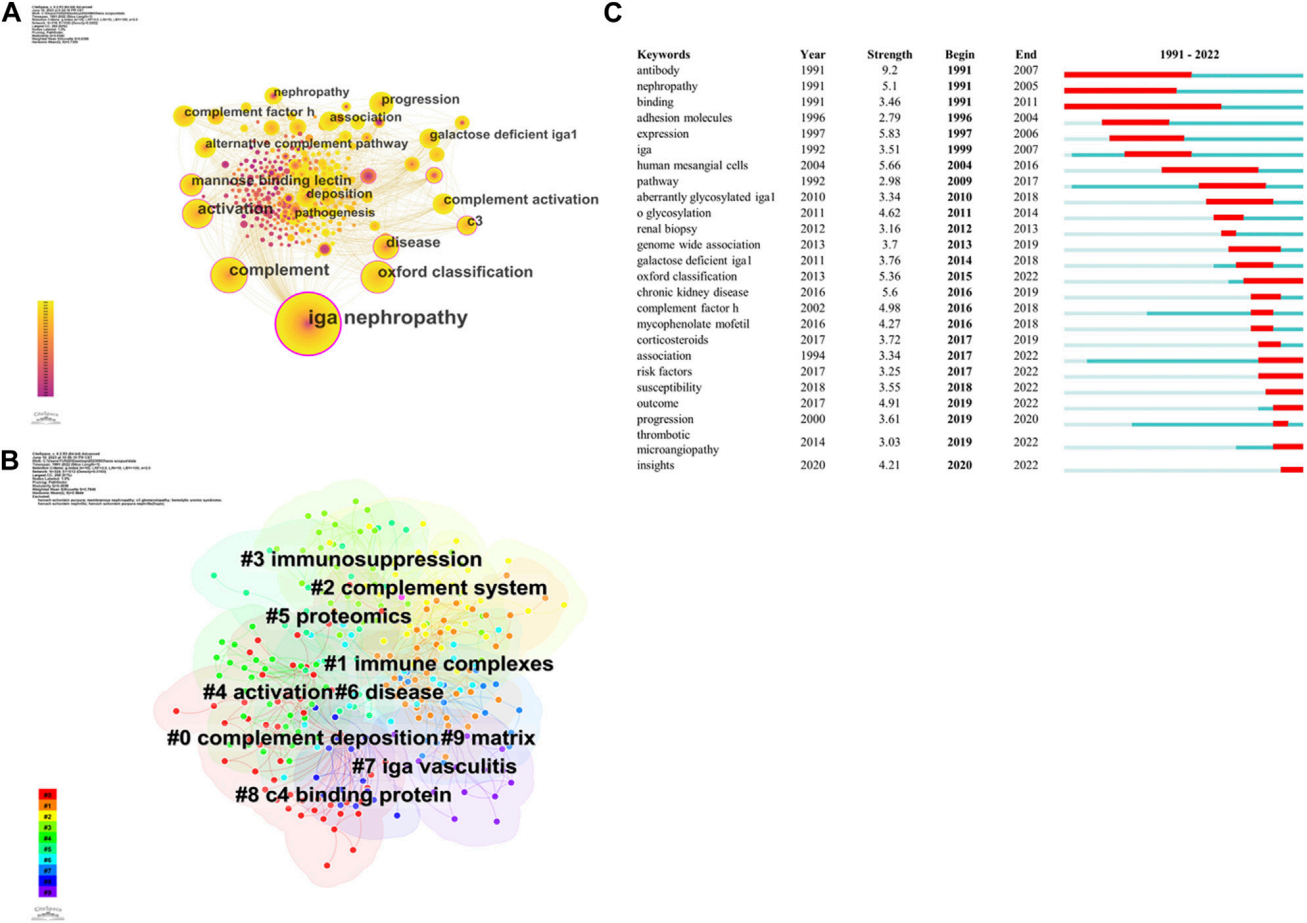
Visualisation of keywords. **(A)** Visualisation of keywords related to complement in IgAN. **(B)** Visualisation of cluster analysis of keywords related to complement in IgAN. **(C)** Visualisation of burst analysis of keywords related to complement in IgAN.

The examination of keywords is instrumental in determining the prevalent research areas and prevailing trends in the field. In this study, the utilization of Citespace software facilitated the identification of a total of 318 keywords, with 208 keywords appearing with a frequency of more than two times (Figure 8A). With regards to the co-occurrence of keywords, there exist keywords that display a centrality value greater than 0.1, among which are iga nephropathy, complement, activation, mannose binding lectin and c3 ect. (Table 6). The glomerular deposition of c3 or c4d is a widely recognized biomarker utilized to indicate the activation of the complement system. Its association with kidney progression in IgAN has been well documented (Zwirner et al., 1997; Jiang et al., 2021). In IgAN, the disease severity is positively correlated with the level of IgA that exposes N-acetyl-d-galactosamine. On the other hand, complement deposits are solely associated with increased levels of IgA1 glycoforms, exhibiting glycan residues with specificity for mannose and N-acetyl-d-glucosamine binding lectins (Medrano et al., 2022). Regarding the role of complement components in IgA nephropathy, we calculated its centrality values:c3 (0.11), complement factor h (0.05), c4 (0.02), anti c1q autoantibody (0), c1q (0), c3a receptor (0), c3b inactivator (0), c3bi receptor (0), c3c (0), c4a (0), c4d (0), c5b-9 (0). C3 has the highest value of centrality (0.11), followed by complement factor h (0.05), c4 (0.02). C3 is the common terminal reaction component of three pathways. About 90% of biopsy samples obtained from patients diagnosed with IgA nephropathy reveal the presence of glomerular co-deposition of c3, in conjunction with immune complexes containing IgA (Medjeral-Thomas et al., 2021). C3aR and C5aR antagonists repressed IgA-induced cell proliferation in cultured human mesangial cells. Moreover, in the IgA nephropathy model induced by Sendai virus, mice deficient in C3aR and C5aR exhibited significant reductions in proteinuria, lower levels of renal IgA and C3 deposition, diminished histological damage, and decreased mesangial proliferation in comparison to wild-type mice (Zhang et al., 2017). Complement factor h is a single-chain glycoprotein that competes with factor B or Bb for binding to C3b, thereby preventing the formation of the C3 convertase in the alternative pathway. The findings from genetic association studies show that deletions in the complement FHR1 and FHR3 genes provide protective effects against IgAN (Gharavi et al., 2011). C4 is a common component of the complement classical pathway and lectin pathway. The presence of c4d deposits in renal biopsies has been found to be an early risk factor for IgAN (Trimarchi and Coppo, 2021). Cluster analysis of keywords was conducted in order to examine the central knowledge framework within the field of study. This study was divided into 10 clusters, as depicted in Figure 8B. Cluster 1 (# 0 complement deposition) comprises 49 keywords, including angiotensin II, c4a. Cluster 2 (# 1 immune complexes) consists 43 keywords, including genome wide association, transferrin receptor. Cluster 3 (#2 complement system)“ has 37 keywords, such as alternative pathway, lectin pathway, kidney disease. Cluster 4 (#3 immunosuppression) has 34 keywords, such as clinical trials, corticosteroids, treatment. Cluster 5 (#4 activation) has 30 keywords, including c3 deficiency, oxford classification, metabolic syndrome. Cluster 6 (#5 proteomics) has 23 keywords, including injury, complement component, pathway. Cluster 7 (#6 disease) has 22 keywords, such as complement component c4, rat, complement factor b. Cluster 8 (#7 iga vasculitis)” has 16 keywords, including mannan binding protein, hypocomplementemia, acute exacerbation. Cluster 9 (#8 c4 binding protein)“has 15 keywords, such as hypocomplementemia, atypical symptoms. Cluster 10 (#9 matrix)” has 15 keywords, such as t-cell/macrophage interaction, chemokines, growth factors. The analysis of the burst of keywords was conducted, and the top 25 keywords with the most powerful citation burst were selected, as illustrated in Figure 8C. Antibody was a prevalent area of investigation within the field of complement research in iga nephropathy during the period from 1991 to 2007.

**TABLE 6:** Top 10 keywords centrality.

Rank	Key word	Centrality
1	iga nephropathy	0.57
2	complement	0.16
3	activation	0.15
4	antibody	0.13
5	disease	0.12
6	mannose binding lectin	0.12
7	expression	0.12
8	c3	0.11
9	oxford classification	0.1
10	complement activation	0.06

## Discussion

### General information

In this study, a bibliometric analysis was conducted on a collection of 819 papers related to complement in IgAN, sourced from the WOS core collection and Scopus database spanning the years 1991–2022. Publications have exhibited a discernible upward trend between 1991 and 2022. In the year 2022, a total of 78 papers were published. Based on current projections, it is anticipated that the quantity of publications in 2023 will exhibit an upward trend in comparison to the previous year. China has emerged as the country with the highest number of publications in this field. The centrality of England was the highest, representing close collaboration between England and other nations. The publications originating from these institutions and authors signify a notable academic reputation for complement in IgAN. The recent surge in national publications attests to the remarkable progress made by researchers in these countries through close collaboration with other countries. Strengthening international cooperation remains imperative to further promote the development of complement in IgAN.

Peking University emerged as the leading institution in terms of publications with a total of 58 papers covering the epidemiology, progression, biomarkers, genetics, and other aspects including crescents and microangiopathic lesions of IgAN (Zhu et al., 2015; Guo et al., 2017; Zhu et al., 2018; Jiang et al., 2021; Li et al., 2022; Wang et al., 2022). The most frequently cited of these was a cross-sectional study and a cohort study by Li Zhu. The study evaluated 1,347 patients with IgAN from Northern China. The result of this study demonstrated that genetic variations in CFH, CFHR3, and CFHR1 can trigger complement activation, which predisposes patients to develop IgAN (Zhu et al., 2015). Additionally, Juntendo University from Japan found that IgAN patients having extraglomerular C3 deposits have worse clinical outcome (Ohsawa et al., 2013).

Professor Hong Zhang of Peking University, and Professor Mohamed R Daha of the University of Groningen are the most prolific contributors in terms of publications and play pivotal leadership roles in the field. Among the top 10 co-cited journals, KI, JASN, and NDT hold a prominent position. The New England Journal of Medicine has the highest impact factor (IF = 176.082) among these journals. JASN and KI are both leading journals in the field of nephrology, publishing both basic and clinical research. However, JASN primarily focuses on clinical research, while KI emphasizes basic research. Recently, KI proposed that circulating complement factor H-related protein 5 level is an independent risk factor for IgAN progression, while JASN reported that the CFHR3,1 Delta genotype did not associate with progression toward CKD stages 3 and 5 in white population of patients with IgAN, although it did associate with a reduced level of glomerular immune deposits. The Nephrology Dialysis Transplantation journal is dedicated to the study of nephrology, dialysis, and transplantation and plays an indispensable role in advancing the care of patients with nephropathy.

The identification of highly cited publications within a period may be a milestone in this field of research. It is notable that Anja Roos from Leiden University Medical Center published an influential publication with 152 citations. The study demonstrated that activation of complement in glomerular is associated with more severe renal disease in IgAN (Roos et al., 2006). Regarding the centrality of co-cited references, the top-ranked publication (0.28) was published by G D’Amico in 1987. This study found that IgA nephropathy is the commonest glomerulonephritis in the world (Damico, 1987).

### Hotspots and trends

Bibliometric studies utilize keywords to reflect the core themes and fundamental content of a publication. By analyzing frequently occurring keywords, one can predict the development of popular topics. Furthermore, the burst of keywords can reveal trends in research hotspots over time. To classify all keywords from the WOS core collection and Scopus database, Citespace was utilized to roughly group them into 10 clusters. Additionally, the burst analysis of keywords was conducted to represent the frontiers and directions of research on complement in IgAN.

**FIGURE 9:**
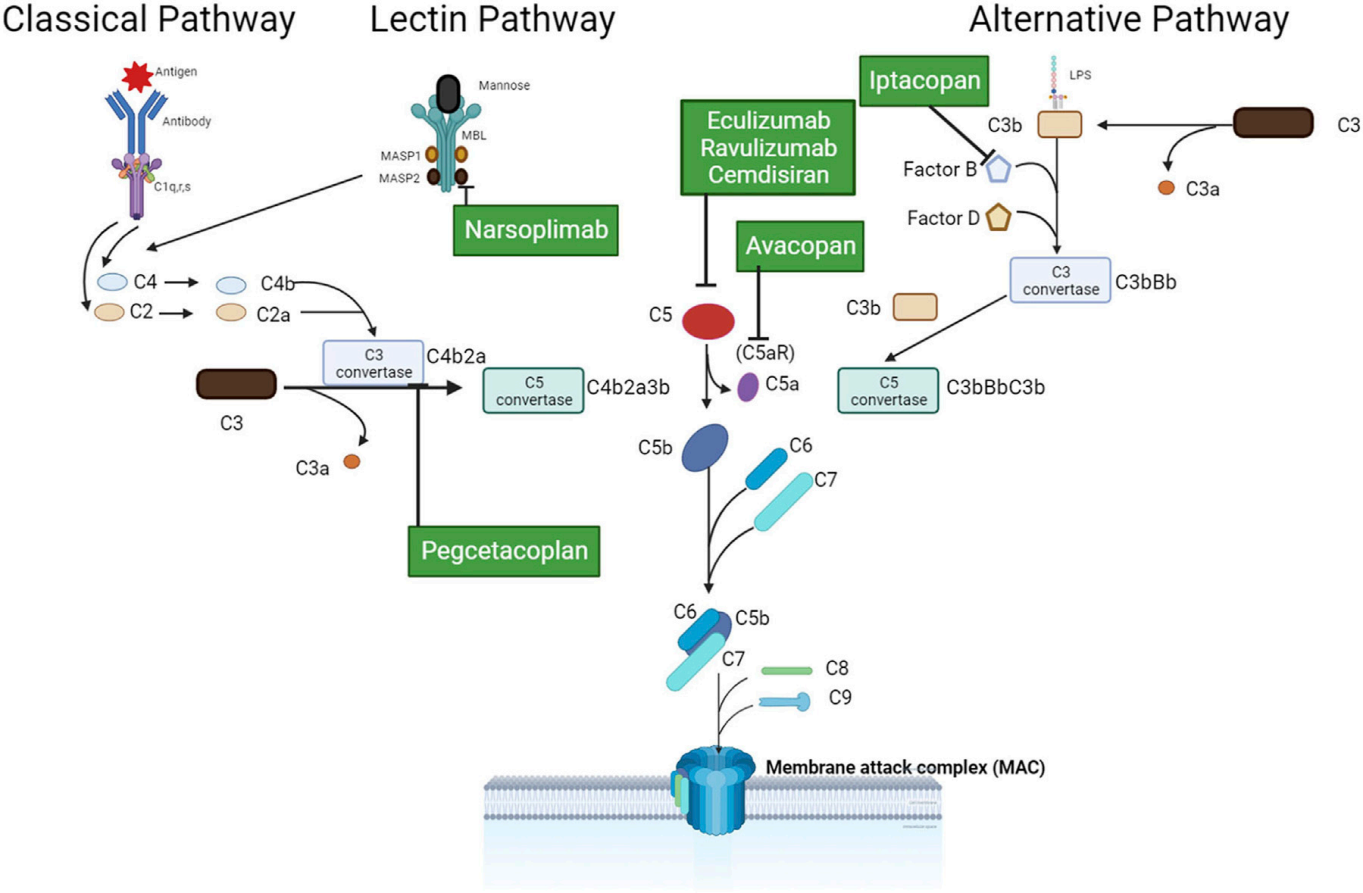
Therapeutic complement inhibitors in IgAN.

The role of complement in the progression of IgA nephropathy is well established, and therapeutic complement inhibitors have been utilized to treat patients with IgAN on a case-by-case basis. Investigational medicinal products targeting terminal complement activation, lectin, and alternative pathway have been used to treat trial participants with IgAN (Figure 9).

Eculizumab is a humanized recombinant monoclonal antibody. It can prevent C5a release, terminal pathway (TP) activation, and the formation of C5b9 by inhibiting C5 convertase activity. Troels Ring reported a 16-year-old male with the vasculitic form of IgAN who failed to respond to aggressive conventional therapy was treated with four weekly doses of 900 mg eculizumab followed by a single dose of 1,200 mg. He responded rapidly to this treatment and has had a stable creatinine (Ring et al., 2015). Therese Rosenblad also found that eculizumab therapy in patients with progressive IgA nephropathy may have a beneficial effect by blocking complement-mediated renal inflammation (Rosenblad et al., 2014). However, A L Herzog presented a case that eculizumab was not effective in treating IGAN recurrence after transplantation (Herzog et al., 2017). Ravulizumab is a humanized, recombinant, long-acting monoclonal antibody that has been designed to target C5 and exhibit effects similar to those of Eculizumab. Currently, it is undergoing evaluation in a phase II clinical trial for the treatment of IgAN (ClinicalTrials.gov Identifier: NCT04564339).

Avacopan (CCX168), a small molecule inhibitor that targets the terminal pathway (TP) of complement by binding the C5a receptor (C5aR), limits the anaphylatoxin and pro-inflammatory effects of C5a. However, unlike eculizumab, it does not affect C5b9 formation. It is plausible that the terminal pathway of complement preserves the critical functions of innate immunity and pathogen defense. An open-label pilot trial was conducted to investigate the effect of avacopan on IgAN in seven patients (Bruchfeld et al., 2022). Notably, proteinuria reduction, an indication of clinical improvement, was achieved in six of the seven participants. However, larger studies with longer follow-up periods are necessary to confirm the efficacy and safety of C5a inhibitors in treating IgAN.

Cemdisiran, also known as ALN-CC5, is a synthetic small interfering RNA (RNAi) that was specifically designed to suppress the production of C5 in the liver. This RNAi has the potential to limit the activation of the terminal complement pathway, which in turn could potentially prevent subsequent inflammation (Rizk et al., 2019). A randomized, placebo-controlled phase II clinical trial is currently underway (ClinicalTrials.gov Identifier: NCT03841448) to evaluate the efficacy and safety of cemdisiran in patients with IgA nephropathy who are experiencing persistent proteinuria greater than 1 g/day despite receiving optimal conservative management.

Narsoplimab (OMS721), a humanized monoclonal antibody to MASP-2 that inhibits lectin pathway (LP) activity, has shown promise in reducing proteinuria in IgAN. In a phase II, multicenter clinical trial, patients diagnosed with IgA nephropathy (IgAN), whose baseline estimated glomerular filtration rate (eGFR) was above 30 mL/min/1.73 m2, and who exhibited proteinuria exceeding 1 g/day despite maximal tolerated renin-angiotensin-aldosterone system (RAAS) blockade, were divided into two sub-studies based on their corticosteroid dependence at baseline. An interim analysis of both groups revealed that the administered drug was found to be safe and well-tolerated, while also effectively reducing proteinuria without adversely impacting eGFR levels (Lafayette et al., 2020). Based on the preliminary data, a phase III clinical trial titled ARTEMIS-IGAN is currently underway to evaluate the efficacy and safety of narsoplimab in IgAN patients with persistent proteinuria greater than 1 g/day, using a randomized, double-blind, placebo-controlled methodology (ClinicalTrials.gov identifier NCT03608033).

Iptacopan (LNP023) is an oral small molecule inhibitor of Factor B, specifically targeting the alternative complement pathway. Results of a recently concluded phase II clinical trial assessing its efficacy in the management of IgAN are eagerly anticipated. Moreover, a phase III clinical trial, named APPLAUSE-IgAN, is currently enrolling participants (ClinicalTrials.gov Identifier: NCT04578834).

Pegcetacoplan (APL-2) has demonstrated the ability to bind with C3 and impede its cleavage into C3a and C3b via C3 convertase (de Castro et al., 2020). Currently, APL-2 is undergoing assessment in a phase II clinical trial as a therapeutic intervention option for patients diagnosed with IgAN and other nephropathy (ClinicalTrials.gov Identifier: NCT03453619).

The complement system is an essential component of the innate immune system, consisting of over 30 components. Abnormal complement activation has been implicated in the pathogenesis of IgAN, but the relationship between each component and IgAN remains unclear. Which component can be used as a biomarker? How to choose the timing of complement targeted treatment is a question worth exploring.

### Limitations

This study represents the first endeavor to visually depict the role of complement in IgAN through the utilization of a bibliometric approach; however, it still has certain limitations. Firstly, it incorporates the WOS core database and Scopus database, other non-English databases such as China National Knowledge Infrastructure (CNKI) are not analyzed. Secondly, this study employed the usage of synonyms during the analysis process, and as a consequence, a certain degree of bias stemming from subjectivity may have been introduced into the results.

## Conclusion

A bibliometric analysis was conducted to examine the research developments, frontiers, and hotspots in complement in IgAN. The findings reveal a rich research background in this field, with a consistent increase in the number of publications over the years. Although a slight decline was observed, it is noteworthy that this research field has continued to stimulate the interest of numerous scholars. The publications evaluated originated from various countries, institutions, authors, and journals, highlighting their significant contributions to the field. Such insights may be leveraged to guide future research. Through an analysis of references and keywords, future research hotspots and trends of complement in IgAN were predicted. For instance, current research hotspots include therapeutic complement inhibitors in IgAN. In conclusion, bibliometrics provides valuable information for the advancement of the challenging research in complement in IgAN.

## Data Availability

The original contributions presented in the study are included in the article/Supplementary material, further inquiries can be directed to the corresponding author.
